# Supplementary material for: Phasic activation of ventral tegmental neurons increases response and pattern similarity in prefrontal cortex neurons
Source: eLife. 2014 Sep 30;3:e02726. doi: 10.7554/eLife.02726 (PMC4206826; doi:10.7554/eLife.02726)
Supplement: Supplementary file 1. — DOI: http://dx.doi.org/10.7554/eLife.02726.011 [file elife02726s001.docx]

**Supplementary file 1**

**ImageJ macros for semi-automatic selecting cells from time series of image files.**

Before starting, two ImageJ plugins should be installed: TurboReg (<http://bigwww.epfl.ch/thevenaz/turboreg/>) and Bio-Formats( <http://loci.wisc.edu/software/bio-formats>).

Move all image files (.oib files) from an animal into a folder named “image”.

My procedures consist of 3 steps. First is aligning the image files using macro1.txt. Second is selecting the regions of interest (ROIs) using macro2.txt. Last is outputting the scores of fluorescent intensities (pixel values) at each ROI using macro3.txt.

**Macro1**

Macro1.txt automatically aligns each image in an .oib file to a target image, which is a Z-projected image of the first .oib file in the “image” folder (Average Intensity). Then, Macro1 creates Z-projected images of aligned-images generated from an .oib file. Now, you should have Z-projected images representing each .oib file, that is, if you have 12 .oib files to analyze, you should have 12 Z-projected images. Macro1 makes a .tiff file named “Z_total_2T1.tiff”, which is a tiff stack file composed of Z-projected images (12 image slices for this example), and stores it in a folder named “Align2T1_Files”.

**Macro2**

Before running Macro2.txt, you may enclose the cells of interest by using the ‘Oval’ tool and ‘ROI Manager’ tool, and saving it as RoiSet in “Align2T1_Files” folder. You should select cells that keep their signal intensity up until the end of the image series.

Above all, Macro2.txt refines the areas of interest (where the cells exist) and saves them as zip files.

**Macro3**

Macro3.txt outputs fluorescence intensities of each cell into a text file.

//Macro1.txt

/////

p0=1; //make ***.tif from ***.oib files.

p1T1=1; //make ***_aligned1.tif (1T1).

p2T1=1; //make ***_aligned2.tif (2T1).

/////

num_data_set=1;

/////////////////////////////////

dir_array = newArray(num_data_set);

for (ii=000; ii<num_data_set; ii++) {

dir_array[ii] = getDirectory("Choose a folder which has oib files");

}

for (ii=000; ii<num_data_set; ii++) {//@@@@@@@

print("start!!");

// "Get Time"

MonthNames = newArray("Jan","Feb","Mar","Apr","May","Jun","Jul","Aug","Sep","Oct","Nov","Dec");

DayNames = newArray("Sun", "Mon","Tue","Wed","Thu","Fri","Sat");

getDateAndTime(year, month, dayOfWeek, dayOfMonth, hour, minute, second, msec);

TimeString ="Date: "+DayNames[dayOfWeek]+" ";

if (dayOfMonth<10) {TimeString = TimeString+"0";}

TimeString = TimeString+dayOfMonth+"-"+MonthNames[month]+"-"+year+"\nTime: ";

if (hour<10) {TimeString = TimeString+"0";}

TimeString = TimeString+hour+":";

if (minute<10) {TimeString = TimeString+"0";}

TimeString = TimeString+minute+":";

if (second<10) {TimeString = TimeString+"0";}

TimeString = TimeString+second;

print(TimeString);

//

dir = dir_array[ii];

dir0=dir;//for batch process

if (p0==1){//***

list = getFileList(dir);

Array.sort(list);

setBatchMode(true);

for (i=000; i<list.length; i++) {

path = dir+list[i];

if (endsWith(list[i], ".oib")){

run("Bio-Formats Importer", "open=["+ path + "] " + "split_channels view=[Standard ImageJ]" + " stack_order=Default " + "series_"+d2s(i+1,0));

dotIndex = lastIndexOf(list[i], ".");

if (dotIndex!=-1)

list[i] = substring(list[i], 0, dotIndex); // remove extension

myDir = dir+"TIFF_Files"+File.separator;

File.makeDirectory(myDir);

sndir = dir+"/TIFF_Files"+"/";

close();

if (nImages==0){

}else{

dotIndex = lastIndexOf(list[i], ".");

if (dotIndex!=-1)

list[i] = substring(list[i], 0, dotIndex);

myDir = dir+"TIFF_Files"+File.separator;

File.makeDirectory(myDir);

sndir = dir+"/TIFF_Files"+"/";

saveAs("Tiff",sndir+list[i]+"_G.tif");

close();

}

if (nImages==0){

}else{

dotIndex = lastIndexOf(list[i], ".");

if (dotIndex!=-1)

list[i] = substring(list[i], 0, dotIndex); // remove extension

myDir = dir+"TIFF_Files"+File.separator;

File.makeDirectory(myDir);

sndir = dir+"/TIFF_Files"+"/";

saveAs("Tiff",sndir+list[i]+"_G.tif");

close();

}

if (nImages==0){

}else{

dotIndex = lastIndexOf(list[i], ".");

if (dotIndex!=-1)

list[i] = substring(list[i], 0, dotIndex);

myDir = dir+"TIFF_Files"+File.separator;

File.makeDirectory(myDir);

sndir = dir+"/TIFF_Files"+"/";

saveAs("Tiff",sndir+list[i]+"_W.tif");

close();

}

}

}

"Done!!0"

// "Get Time"

MonthNames = newArray("Jan","Feb","Mar","Apr","May","Jun","Jul","Aug","Sep","Oct","Nov","Dec");

DayNames = newArray("Sun", "Mon","Tue","Wed","Thu","Fri","Sat");

getDateAndTime(year, month, dayOfWeek, dayOfMonth, hour, minute, second, msec);

TimeString ="Date: "+DayNames[dayOfWeek]+" ";

if (dayOfMonth<10) {TimeString = TimeString+"0";}

TimeString = TimeString+dayOfMonth+"-"+MonthNames[month]+"-"+year+"\nTime: ";

if (hour<10) {TimeString = TimeString+"0";}

TimeString = TimeString+hour+":";

if (minute<10) {TimeString = TimeString+"0";}

TimeString = TimeString+minute+":";

if (second<10) {TimeString = TimeString+"0";}

TimeString = TimeString+second;

print(TimeString);

//

}//***

if (p1T1==1){//***

dir=dir0+"TIFF_Files"+File.separator;

list = getFileList(dir);

Array.sort(list);

setBatchMode(true);

for (i=000; i<list.length; i++) {

path = dir+list[i];

if (endsWith(list[i], "/")){

//if ends with "/", do nothing and skip to the next file

}else if(endsWith(list[i], "_G.tif")){

/////////

run("Bio-Formats Importer", "open=["+ path + "] " + "split_channels view=[Standard ImageJ]" + " stack_order=Default " + "series_"+d2s(i+1,0));

s_num=nSlices;

title1=getTitle();

imgid1=getImageID();

bitdepth=bitDepth();

height=getHeight();

width=getWidth();

dotIndex = lastIndexOf(list[i], ".");

if (dotIndex!=-1)

list[i] = substring(list[i], 0, dotIndex); // remove extension

bs_index=lastIndexOf(dir, "\\");//one back slash"\" is not recognized then I used "\\"

dir2=substring (dir, 0, bs_index);

bs_index=lastIndexOf(dir2, "\\");

dir2=substring(dir2, 0, bs_index);

myDir = dir2+File.separator+"Align1T1_Files"+File.separator;

File.makeDirectory(myDir);

sndir = dir2+"/Align1T1_Files"+"/";

///////////////////////////

new_name=list[i]+ "_aligned1";

// this stack is for aligned file.

run("Duplicate...", "title=" + new_name + "duplicate range=1-1");

dstId = getImageID();

run("Add Slice");

setSlice(2);

run("32-bit");

height=getHeight();

width=getWidth();

//make reference image.

selectWindow(title1);

run("Duplicate...", "title=ref duplicate range=1-1");

imgid_ref = getImageID();

dstId_ref = getTitle();

//make a blank image to paste the target image.

run("Duplicate...", "title=target duplicate range=1-1");

imgid_tar = getImageID();

dstId_tar = getTitle();

for (j=2 ;j<=s_num ;j++){

jj=j+1;

selectWindow(title1);

setSlice(j);

run("Select All");

run("Copy");

selectImage(imgid_tar);

run("Paste");

rename(dstId_tar);

run("TurboReg ",

"-align " //

+ "-window " + dstId_tar + " "//

+ "0 0 " + (width - 1) + " " + (height - 1) + " " //

+ "-window " + dstId_ref + " "// reference file.

+ "0 0 " + (width - 1) + " " + (height - 1) + " " //

+ "-rigidBody " //

+ (width / 2) + " " + (height / 2) + " " //

+ (width / 2) + " " + (height / 2) + " " //

+ "0 " + (height / 2) + " " //

+ "0 " + (height / 2) + " " //

+ (width - 1) + " " + (height / 2) + " " //

+ (width - 1) + " " + (height / 2) + " " //

+ "-showOutput"); //

selectWindow("Output");

rename("registered");

setSlice(1);

run("Select All");

run("Copy");

selectImage(dstId);

run("Paste");

run("Add Slice");

setSlice(j+1);

selectWindow("registered");

close();

}

selectImage(dstId);

run("Delete Slice");

/////////////////////////////////////

saveAs("Tiff",sndir+new_name+".tif");

rename(dstId);

run("Z Project...", "start=1 stop=" + s_num + " projection=[Average Intensity]");

saveAs("Tiff",sndir+list[i]+"_1Z.tif");

close();

selectImage(dstId);

close();

selectWindow(dstId_tar);

close();

selectWindow(dstId_ref);

close();

selectWindow(title1);

close();

}

}

//////////////////////////////////////////////////

//Make a stack from all Z projected images.

///////////////////////////////////////////////////

list = getFileList(sndir);

Array.sort(list);

flag=0;

jjj=0;

for (i=000; i<list.length; i++) {//

path = sndir+list[i];

if (endsWith(list[i], "/")){

//if ends with "/", do nothing and skip to the next file

}else if(endsWith(list[i], "Z.tif") && flag==0){

label_name=list[i];

label_name=substring(label_name, 0, 9);

// run("Bio-Formats Importer", "open=["+ path + "] " + "split_channels view=[Standard ImageJ]" + " stack_order=Default " + "series_"+d2s(i+1,0));

open(path);

Z_title=getTitle();

setMetadata("Label", label_name);

jjj=1;

flag=1;

}else if (endsWith(list[i], "Z.tif") && flag==1){

label_name=list[i];

label_name=substring(label_name, 0, 9);

open(path);

run("Select All");

run("Copy");

close();

selectImage(Z_title);

setSlice(jjj);

run("Add Slice");

run("Paste");

setMetadata("Label", label_name);

jjj=jjj+1;

}

}

selectImage(Z_title);

saveAs("Tiff",sndir+"Z_total_1T1.tif");

close();

"Done1T1!!"

// "Get Time"

MonthNames = newArray("Jan","Feb","Mar","Apr","May","Jun","Jul","Aug","Sep","Oct","Nov","Dec");

DayNames = newArray("Sun", "Mon","Tue","Wed","Thu","Fri","Sat");

getDateAndTime(year, month, dayOfWeek, dayOfMonth, hour, minute, second, msec);

TimeString ="Date: "+DayNames[dayOfWeek]+" ";

if (dayOfMonth<10) {TimeString = TimeString+"0";}

TimeString = TimeString+dayOfMonth+"-"+MonthNames[month]+"-"+year+"\nTime: ";

if (hour<10) {TimeString = TimeString+"0";}

TimeString = TimeString+hour+":";

if (minute<10) {TimeString = TimeString+"0";}

TimeString = TimeString+minute+":";

if (second<10) {TimeString = TimeString+"0";}

TimeString = TimeString+second;

print(TimeString);

//

}//***

/////////////////////////////////////////

//////////////////////////////////////////

if(p2T1==1){//***

dir = dir0+"Align1T1_Files"+File.separator;

list = getFileList(dir);

Array.sort(list);

setBatchMode(true);

flag=0;

for (i=000; i<list.length; i++) {

path = dir+list[i];

if (endsWith(list[i], "/")){

//if ends with "/", do nothing and skip to the next file

}else if(flag==0 && endsWith(list[i], "Z.tif")){

open(path);

ref0_title=getTitle();

ref0_imgid=getImageID();

flag=1;

}

}

for (i=000; i<list.length; i++) {

path = dir+list[i];

if (endsWith(list[i], "/")){

//if ends with "/", do nothing and skip to the next file

}

else if(endsWith(list[i], "aligned1.tif")){

run("Bio-Formats Importer", "open=["+ path + "] " + "split_channels view=[Standard ImageJ]" + " stack_order=Default " + "series_"+d2s(i+1,0));

s_num=nSlices;

title1=getTitle();

imgid1=getImageID();

bitdepth=bitDepth();

height=getHeight();

width=getWidth();

selectWindow(title1);

run("Z Project...", "start=1 stop=" + s_num + " projection=[Average Intensity]");

rename("tar0");

tar0_title=getTitle();

run("32-bit");

run("TurboReg ",

"-align " //

+ "-window " + "tar0" + " "//

+ "0 0 " + (width - 1) + " " + (height - 1) + " " //

+ "-window " + ref0_title + " "//

+ "0 0 " + (width - 1) + " " + (height - 1) + " " //

+ "-rigidBody " //

+ (width / 2) + " " + (height / 2) + " " //

+ (width / 2) + " " + (height / 2) + " " //

+ "0 " + (height / 2) + " " //

+ "0 " + (height / 2) + " " //

+ (width - 1) + " " + (height / 2) + " " //

+ (width - 1) + " " + (height / 2) + " " //

+ "-showOutput"); //

selectWindow("Output");

rename("2nd_ref");

setSlice(2);

run("Delete Slice");

imgid_ref = getImageID();

dstId_ref = getTitle();

dotIndex = lastIndexOf(list[i], "_");

if (dotIndex!=-1)

list[i] = substring(list[i], 0, dotIndex); // remove extension

bs_index=lastIndexOf(dir, "\\");//one back slash"\" is not recognized then I used "\\"

dir2=substring (dir, 0, bs_index);

bs_index=lastIndexOf(dir2, "\\");

dir2=substring(dir2, 0, bs_index);

myDir = dir2+File.separator+"Align2T1_Files"+File.separator;

File.makeDirectory(myDir);

sndir = dir2+"/Align2T1_Files"+"/";

///////////////////////////

new_name=list[i]+ "_aligned2";

// this stack is for aligned file.

selectWindow(title1);

run("Duplicate...", "title=" + new_name + "duplicate range=1-1");

dstId = getImageID();

run("32-bit");

height=getHeight();

width=getWidth();

//make a blank image to paste the target image.

selectWindow(title1);

run("Duplicate...", "title=target duplicate range=1-1");

imgid_tar = getImageID();

dstId_tar = getTitle();

for (j=1 ;j<=s_num ;j++){

jj=j+1;

selectWindow(title1);

setSlice(j);

run("Select All");

run("Copy");

selectImage(imgid_tar);

run("Paste");

rename(dstId_tar);

run("TurboReg ",

"-align " //

+ "-window " + dstId_tar + " "//

+ "0 0 " + (width - 1) + " " + (height - 1) + " " //

+ "-window " + dstId_ref + " "//

+ "0 0 " + (width - 1) + " " + (height - 1) + " " //

+ "-rigidBody " //

+ (width / 2) + " " + (height / 2) + " " //

+ (width / 2) + " " + (height / 2) + " " //

+ "0 " + (height / 2) + " " //

+ "0 " + (height / 2) + " " //

+ (width - 1) + " " + (height / 2) + " " //

+ (width - 1) + " " + (height / 2) + " " //

+ "-showOutput"); //

//

selectWindow("Output");

rename("registered");

setSlice(1);

run("Select All");

run("Copy");

selectImage(dstId);

run("Paste");

run("Add Slice");

setSlice(j+1);

selectWindow("registered");

close();

}

selectImage(dstId);

run("Delete Slice");

/////////////////////////////////////

saveAs("Tiff",sndir+new_name+".tif");

rename(dstId);

run("Z Project...", "start=1 stop=" + s_num + " projection=[Average Intensity]");

saveAs("Tiff",sndir+list[i]+"_2Z.tif");

close();

selectImage(dstId);

close();

selectWindow(dstId_tar);

close();

selectWindow(dstId_ref);

close();

selectWindow(title1);

close();

selectWindow(tar0_title);

close();

}

}

selectWindow(ref0_title);

close();

//////////////////////////////////////////////////

//Make a stack from all Z projected images.

///////////////////////////////////////////////////

list = getFileList(sndir);

Array.sort(list);

flag=0;

jjj=0;

for (i=000; i<list.length; i++) {/

path = sndir+list[i];

if (endsWith(list[i], "/")){

//if ends with "/", do nothing and skip to the next file

}else if(endsWith(list[i], "Z.tif") && flag==0){

label_name=list[i];

label_name=substring(label_name, 0, 9);

open(path);

Z_title=getTitle();

setMetadata("Label", label_name);

jjj=1;

flag=1;

}else if (endsWith(list[i], "Z.tif") && flag==1){

label_name=list[i];

label_name=substring(label_name, 0, 9);

open(path);

run("Select All");

run("Copy");

close();

selectImage(Z_title);

setSlice(jjj);

run("Add Slice");

run("Paste");

setMetadata("Label", label_name);

jjj=jjj+1;

}

}

selectImage(Z_title);

saveAs("Tiff",sndir+"Z_total_2T1.tif");

close();

"Done!!2T1"

// Get Time

MonthNames = newArray("Jan","Feb","Mar","Apr","May","Jun","Jul","Aug","Sep","Oct","Nov","Dec");

DayNames = newArray("Sun", "Mon","Tue","Wed","Thu","Fri","Sat");

getDateAndTime(year, month, dayOfWeek, dayOfMonth, hour, minute, second, msec);

TimeString ="Date: "+DayNames[dayOfWeek]+" ";

if (dayOfMonth<10) {TimeString = TimeString+"0";}

TimeString = TimeString+dayOfMonth+"-"+MonthNames[month]+"-"+year+"\nTime: ";

if (hour<10) {TimeString = TimeString+"0";}

TimeString = TimeString+hour+":";

if (minute<10) {TimeString = TimeString+"0";}

TimeString = TimeString+minute+":";

if (second<10) {TimeString = TimeString+"0";}

TimeString = TimeString+second;

print(TimeString);

//

}//***

"Done!!"

//Macro2.txt

//set parameter*************

pick_rate=0.95; // The ROI is set at the border whose intensity levels are more than “pick_rate” (in this case more than 0.95% of the center of each selected oval area).

//*********************************

//dir = getDirectory("Choose Align2T1_Files");

dir = getDirectory("Choose a folder named Align2T1_Files");

list = getFileList(dir);

Array.sort(list);

// setBatchMode(true);

aling_tif="_aligned2.tif";

Z_total_tif="Z_total_2T1.tif";

n1=0;

for (i=000; i<list.length; i++) {

if (endsWith(list[i], aling_tif)){

n1=n1+1;

}

}

n2=0;

filelist1=newArray(n1);

for (i=000; i<list.length; i++) {

if (endsWith(list[i], aling_tif)){

filelist1[n2]=list[i];

n2=n2+1;

}

}

Array.sort(filelist1);

savelist=newArray(n1);

for (i=000; i<filelist1.length; i++) {

dotIndex = lastIndexOf(filelist1[i], "_");

savelist[i] = substring(filelist1[i], 0, dotIndex); // remove extension

new_name=savelist[i]+ "_roi.zip";

print(savelist[i]);

}

path = dir+"RoiSet.zip";

open(path);

roiManager("Delete");

setBatchMode(true);

path1 = dir+Z_total_tif;

open(path1);

//do the Gaussian filter on the Z_total2Tmax.tif

selectWindow(Z_total_tif);

s_num=nSlices;

run("Duplicate...", "title=Z_total_roi.tif duplicate range=1-" + s_num );

roin=nSlices;

for (n=0; n<roin; n++) {

selectWindow("Z_total_roi.tif");

setSlice(roin-n);

run("Copy");

run("Add Slice");

run("Paste");////add a dummy slice which is going to be deleted later.

}

selectWindow(Z_total_tif);

run("Gaussian Blur...", "sigma=3 stack");

run("Copy");

run("Internal Clipboard");

selectWindow("Clipboard");// this is named as "Clipboard"

changeValues(0, 100000, 0);

////each task for each stack.

selectWindow(Z_total_tif);

nsl=nSlices;

setSlice(nsl);

run("Add Slice");

for (n=1; n<=nsl; n++) {///############

open(path);

roiManager("Select", 0);

roiManager("Add");//add the dummy roi which is deleted later.

roi_c=roiManager("count")-1;

for (i=000; i<roi_c+1; i++) {//@@@@@@@@

selectWindow(Z_total_tif);

run("Select All");

run("Copy");

run("Internal Clipboard");

rename("Clipboard-2");

selectWindow("Clipboard");

run("Copy");

run("Internal Clipboard");

rename("Clipboard-1");

selectWindow(Z_total_tif);

setSlice(1);

roiManager("Select", i);

run("Copy");

selectWindow("Clipboard-1");

roiManager("Select", i);

run("Paste");

getSelectionBounds(xx, yy, xwidth, yheight);

//*********find peak and store the location

selectWindow("Clipboard-1");

width = getWidth;

height = getHeight;

countmax=0; xmax=0; ymax=0;

getSelectionBounds(x, y, width1, height1);

xmax=round(x+width1/2);

ymax=round(y+height1/2);

countmax=getPixel(xmax,ymax);

for(x = xx ; x <= xx + xwidth;x++){

for(y=yy;y<=yy+yheight;y++){

if(pick_rate*countmax<getPixel(x,y)){

selectWindow("Z_total_roi.tif");

setSlice(2*n-1);

setPixel(x,y,0);

selectWindow("Clipboard-2");

setPixel(x,y,0);

selectWindow("Clipboard-1");

}

}

}

selectWindow("Clipboard-1");

close();

//********

selectWindow("Clipboard-2");

roiManager("Select", i);

makeOval(0, 0, 2, 1);//this oval is a dummy

doWand(xmax, ymax);

roiManager("Update");

selectWindow("Clipboard-2");

close();

}//@@@@@@@@@

selectWindow(Z_total_tif);

rename(Z_total_tif);

run("Delete Slice");

print(nsl-n);

roiManager("Delete");//delete dummy roi.

roiManager("Save", dir+ savelist[n-1]+ "_roi.zip");

//roiManager("Save", dir+ n+ "_roi.zip");

roiManager("Delete");

}///############

selectWindow("Z_total_roi.tif");

saveAs("Tiff",dir+"Z_total_2Tmax_roi.tif");

"Done!!"

//Macro3.txt

dir = getDirectory("Choose a folder named Align2T1_Files");

list = getFileList(dir);

Array.sort(list);

setBatchMode(true);

aling_tif="_aligned2.tif";

n1=0;

for (i=000; i<list.length; i++) {

if (endsWith(list[i], aling_tif)){

n1=n1+1;

}

}

n2=0;

filelist1=newArray(n1);

filelist2=newArray(n1);

for (i=000; i<list.length; i++) {

if (endsWith(list[i], aling_tif)){

filelist1[n2]=list[i];

n2=n2+1;

}

}

nn=0;

for (i=000; i<list.length; i++) {

if (endsWith(list[i], "_roi.zip")){

filelist2[nn]=list[i];

nn=nn+1;

}

}

Array.sort(filelist1);

Array.sort(filelist2);

savelist=newArray(n1);

for (i=000; i<filelist1.length; i++) {

dotIndex = lastIndexOf(filelist1[i], "_");

savelist[i] = substring(filelist1[i], 0, dotIndex); // remove extension

new_name=savelist[i]+ "_oib.txt";

print(savelist[i]);

}

run("ROI Manager...");

setBatchMode(true);

for (k=0; k<nn; k++) {///############

path = dir+filelist1[k];

open(path);

rename(filelist1[k]);

num_s=nSlices;

path = dir+filelist2[k];

open(path);

roiManager("Multi Measure");

saveAs("Results", dir + savelist[k]+ "_oib.txt");

roiManager("Delete");

selectWindow(filelist1[k]);

close();

run("Clear Results");

i=nn-k;

print ( i );

}

"Done!!"
